# Sequence analysis of Epstein-Barr virus EBNA-2 gene coding amino acid 148-487 in nasopharyngeal and gastric carcinomas

**DOI:** 10.1186/1743-422X-9-49

**Published:** 2012-02-21

**Authors:** Xinying Wang, Yun Wang, Guocai Wu, Yan Chao, Zhifu Sun, Bing Luo

**Affiliations:** 1 Department of Medical Microbiology, Qingdao University Medical College, 38 Dengzhou Road, Qingdao, 266021, China; 2 Department of Health Sciences Research, Mayo Clinic, Rochester, Minnesota, USA; 3 Department of Clinical Laboratory, Guangdong Provincial Hospital of Chinese Medicine, 111 Dade Road, Guangzhou, 510120, China

**Keywords:** Epstein-Barr virus, Gastric carcinoma, Nasopharyngeal carcinoma, Nuclear antigen 2, Polymorphism

## Abstract

**Background:**

The Epstein-Barr virus (EBV) nuclear antigen 2 (EBNA-2) plays a key role in the B-cell growth transformation by initiating and maintaining the proliferation of infected B-cell upon EBV infection in vitro. Most studies about EBNA-2 have focused on its functions yet little is known for its intertypic polymorphisms.

**Results:**

Coding region for amino acid (aa) 148-487 of the EBNA-2 gene was sequenced in 25 EBV-associated gastric carcinomas (EBVaGCs), 56 nasopharyngeal carcinomas (NPCs) and 32 throat washings (TWs) from healthy donors in Northern China. Three variations (g48991t, c48998a, t49613a) were detected in all of the samples (113/113, 100%). EBNA-2 could be classified into four distinct subtypes: E2-A, E2-B, E2-C and E2-D based on the deletion status of three aa (294Q, 357K and 358G). Subtypes E2-A and E2-C were detected in 56/113 (49.6%), 38/113 (33.6%) samples, respectively. E2-A was observed more in EBVaGCs samples and subtype E2-D was only detected in the NPC samples. Variation analysis in EBNA-2 functional domains: the TAD residue (I438L) and the NLS residues (E476G, P484H and I486T) were only detected in NPC samples which located in the carboxyl terminus of EBNA-2 gene.

**Conclusions:**

The subtypes E2-A and E2-C were the dominant genotypes of the EBNA-2 gene in Northern China. The subtype E2-D may be associated with the tumorigenesis of NPC. The NPC isolates were prone harbor to more mutations than the other two groups in the functional domains.

## Background

Epstein-Barr virus (EBV) is a ubiquitous herpes virus infecting the majority of human populations. Its genome approximately has 172,000 base pairs [1]. EBV plays an important role in various human tumors, such as Burkitt's lymphoma (BL), nasopharyngeal carcinoma (NPC) [2-4] and causes the benign lymphoproliferative disease infectious mononucleosis [5]. It is also associated with 10% of gastric carcinomas (GC) [6,7], often called EBV-associated gastric carcinoma (EBVaGC). In Northern China this rate is about 7.0% according to our previous study [8].

In vitro, EBV can latently infect and immortalize human B lymphocytes. EBNA-LP and EBNA-2 are firstly expressed viral genes, followed by the other latency genes EBNA-1, EBNA-3A, EBNA-3B, EBNA-3C, latent membrane protein (LMP) -1, LMP-2 and the small nonpolyadenylated RNAs (EBERs) [9-12]. The role of EBNA-2 in B-cell growth transformation is closely linked to transactivation of cellular and viral gene expression. The expression of the LMP genes and B-cell genes, including CD23, CD21 and c-fgr are transactivated by EBNA-2 [13]. By activating viral as well as cellular target genes, EBNA-2 initiates the transcription of a cascade of primary and secondary target genes, which eventually govern the activation of the resting B-cell, cell cycle entry and proliferation of the growth transformed cells.

EBV isolates are classified as EBV type A and B (also known as type 1 and 2) according to the sequence of the EBNA-2 gene. EBV-A shows more efficient transforming activity in vitro than EBV-B type, and is predominantly found in EBV-associated diseases [14]. The amino acid sequences of EBNA-2 in EBV type A and B are remarkably divergent. This divergence is potentially useful for predicting essential domains of the protein. The protein consists of a lightly negatively charged, well-conserved amino terminus (aa 1 to 58); a polyproline domain which differs in length (aa 59 to 95); a short conserved charged domain (aa 96 to 134); a long divergent domain (aa 135 to 281); a moderately well conserved proline-rich domain (aa 282 to 330); a moderately well conserved basic domain, including an arginine-glycine repeat motif (aa 331 to 369); a moderately well conserved acidic domain (aa 370 to 475); and a short, basic, conserved carboxyl terminus (aa 476 to 487) [15]. As a transcription factor, EBNA-2 contains three domains critical for its transcription regulation function, the self-association domain (aa 101-214), transactivation domain (TAD) (aa 424-468), and nuclear localization signals (NLS) (aa 284-341 and aa 468-487) [4,16,17]. We hypothesized that sequence variations in this region more likely affect its regulation function and therefore are possibly related to the development of malignancy.

Most studies on sequence variations of the EBV genome have focused on the populations in Southern China, the endemic area of NPC [18-22]. However, the nucleotide sequence of EBV type A and B is different extensively and consistently in EBNA-2, -3A, -3B and -3C genes [23]. No report has been available for intertypic polymorphisms of EBNA-2 gene in EBV-positive NPCs and EBVaGCs samples in China. This study analyzed the key nucleotide sequences of EBNA-2 gene that codes aa from 148 to 487 and determined the variations of this gene in EBVaGCs, NPCs and TWs in Northern China, a non-NPC endemic region.

## Results

### Sequence variation of EBNA-2 gene

**Figure 1:**
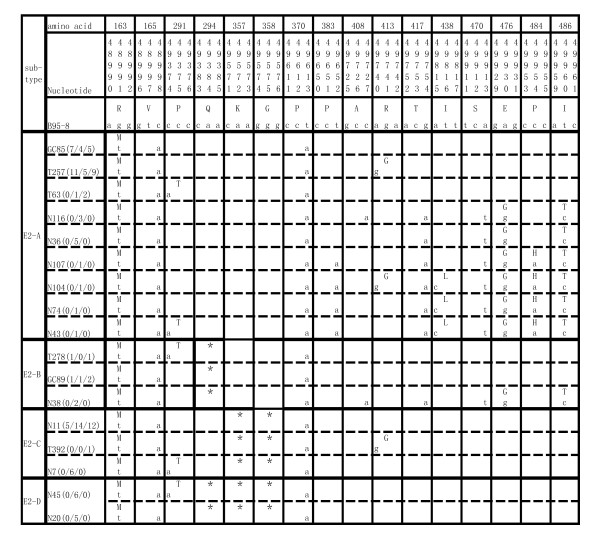
**Aa 148-487 of the EBNA-2 gene sequence variations in EBVaGC, NPC biopsies and TWs of healthy donors in Northern China**. The aa (148-487) and their corresponding nucleotide sequence variations of the EBNA-2 in the EBVaGC, NPC and TW from healthy donors in Northern China. Numbers on the top correspond to the aa and nucleotide positions of the gene under which the B95-8 prototype aa and nucleotide sequences are listed. Different subtypes are noted on the left of the table, while the specimens showing identical sequences to each other are represented by a sample in that group listed in the second column (such as GC85, T257...). The number in the parenthesis separated by "/" denotes the number of sample isolates with the identical sequence from EBVaGC, NPC and TW, respectively. '*' stands for the deletion of an aa. The previously reported mutations at positions 49102 and 49136 were not detected in isolates from our area. A 51bp deletion was at nucleotide 49102 and a triplet insertion of ctc was at nucleotide 49136 [29].

The sequences of EBNA-2 gene for aa 148-487 were determined in 25 EBVaGCs, 56 NPCs and 32 TWs samples by PCR sequencing. All the sequences were compared with the prototype B95-8 sequence. Nucleotide changes were detected in 20 loci, 10 of which resulted in aa changes (Figure 1). Among the 20 loci with nucleotide changes, three nucleotide mutations (g48991t, c48998a and t49613a) were detected in all the EBVaGC, NPC and TW samples (113/113, 100%) (Figure 1). According to whether there was a deletion in the sequences, 4 distinct subtypes of EBNA-2 were classified, namely subtype E2-A (no aa deletion), E2-B (aa 294Q deletion), E2-C (aa 357K, 358G deletion), and E2-D (aa 294Q, aa 357K and 358G deletion). Two subtypes, E2-A and E2-C, were found to be dominant in the total specimens. The E2-D pattern was only detected in the NPC samples (Table 1).

**Table 1:** Distribution of EBNA-2 gene subtypes (aa 148-487) in the EBVaGC, NPC and TWs

Subtype	EBVaGC (n = 25)	NPC (n = 56)	TWs (n = 32)	Total (n = 113)
E2-A	18 (72%)	22 (39.3%)	16 (50%)	56 (49.6%)
E2-B	2 (8%)	3 (5.4%)	3 (9.4%)	8 (7.1%)
E2-C	5 (20%)	20 (35.7%)	13 (40.6%)	38 (33.6%)
E2-D	0	11 (19.6%)	0	11 (9.7%)

EBVaGCs, EBV-associated gastric carcinomas; NPC, nasopharyngeal carcinomas; TWs, throat washings from healthy donors.

Fifty-six of 113 samples (49.6%) were detected in subtype E2-A (Table 1). This type had six non-silent mutations (P291T, R413G, I438L, E476G, P484H and I486T) and four silent mutations (383cct-cca, 408gcc-gca, 417acg-aca and 470tca-tct) in addition to the three common mutations (Figure 1). Interestingly, the four silent mutations and five non-silent mutations (R413G, I438L, E476G, P484H and I486T) in this subtype were only detected in 12 NPC samples. All these mutations are located in the EBNA-2 acidic domain.

The second common subtype E2-C was detected in 33.6% (38/113) of the tested samples, which contained two aa (357K, 358G) deletions (Table 1). Residue P291T were present in 6 NPC samples and the residue R413G was detected only in TW392.

The subtype E2-B was detected in 8 samples with the common deletion of aa 294Q. Interestingly, subtype E2-D (aa 294Q, 357K, 358G deletion) was only detected in the NPC samples. The residue P291T mutation existed in 6 NPC samples (Figure 1).

### Distribution of EBNA2 gene subtypes in EBVaGCs, NPCs, and TWs

The frequency of EBNA-2 subtypes in EBVaGCs, NPCs and TWs of healthy donors was summarized in Table 1. Fisher's exact test was used to determine the difference of the EBNA2 subtypes among the EBVaGCs, NPCs and the TWs. E2-A and E2-C were dominant subtypes in the tested specimens. E2-A was detected in 49.6% (56/113) of total specimens, 18/25(72%) in EBVaGCs, 22/56 (39.3%) in NPCs, and 16/32 (50%) in TWs. E2-C was found in 33.6% (38/113) specimens, including 5/25 (20%) EBVaGCs, 20/56(35.7%) NPCs, and 13/32 (40.6%) TWs (Table 1). The proportion of the E2-A in EBVaGCs (72%, 18/25) was significantly higher than in NPC (39.3%, 22/56) or TWs (50%, 16/32). The E2-D subtype was only detected in the NPC samples (Table 1) (*P *= 0.008).

### Variation analysis in EBNA-2 functional domains

**Figure 2:**
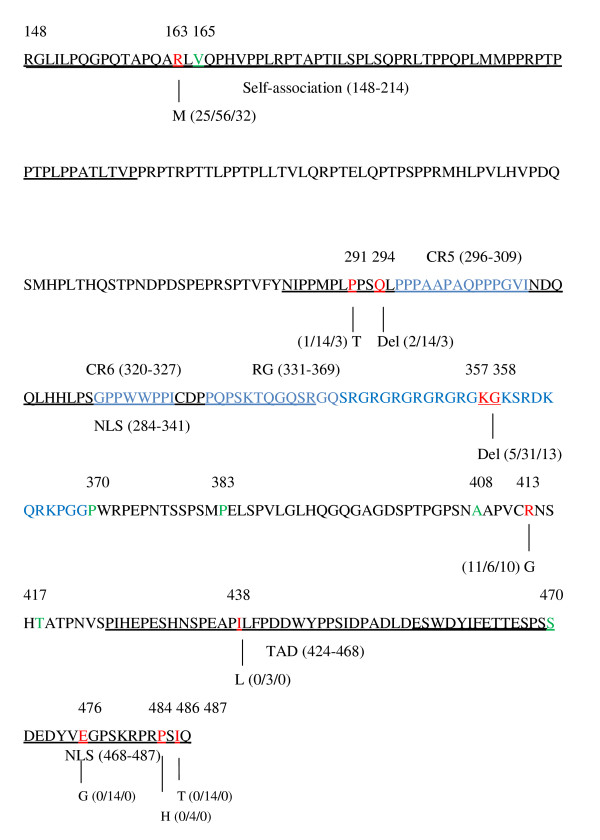
**Amino acid sequence of the aa 148-487 in EBNA-2 gene and the mutated amino acids in isolates from EBVaGCs, NPCs and TWs in Northern China**. Schematic illustration of the aa 148-487 of EBNA-2. The functional motifs (self-associational domain, NLS, RG domain and TAD) are underlined. Numbers indicate aa positions. The silent mutated aa positions are shown in green color and non-silent mutated sequences are in red color. The following numbers separated by "/" denote the number of the identical sequences from EBVaGC, NPC and TWs, respectively. The blue alphabets represent CR5, CR6 and RG regions of EBNA-2, respectively. The "Del" indicates the deletion of the aa.

**Figure 3:**
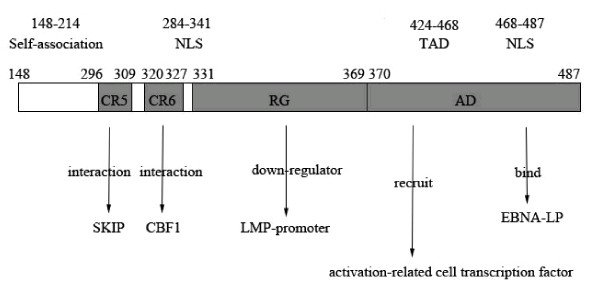
**The schematic model of aa 148-487 EBNA-2 involved in functional domains**. Schematic aa 148-487 of EBNA-2 illustrating the relation of functional domains in this study. The aa number of self-association domain, CR5, CR6, NLS, RG, AD (TAD and NLS) are indicated. And the CR5, CR6, RG, AD domains are represented by ashy rectangle.

EBNA-2 carries the characteristic features common to all transcription factors: TAD, NLS and a region which mediates promoter contact (Figure 2 and 3). The aa mutations in EBNA-2 functional domains are summarized in Table 2. The mutation at residue R163M was detected in all samples in the self-association domain. Interestingly, the mutations in TAD residue (I438L) and the NLS residues (E476G, P484H and I486T) were only detected in NPC samples which located in the carboxyl terminus of EBNA-2 gene. Furthermore, the other functional domains of EBNA-2 gene were detected to have more aa mutations in NPC samples.

**Table 2:** Distribution of aa mutations in EBNA-2 functional domains

Functional domains	Residues	EBVaGC(n = 25)	NPC(n = 56)	TWs(n = 32)
Self-association	163(R-M)	25(100%)	56(100%)	32(100%)
NLS	291(P-T)	1(4%)	14(25%)	3(9.4%)
	294(Qdel)	2(8%)	14(25%)	3(9.4%)
RG domain	357(Kdel)	5(20%)	31(55.4%)	13(40.6%)
	358(Gdel)	5(20%)	31(55.4%)	13(40.6%)
TAD	438(I-L)	0	3(5.4%)	0
NLS	476(E-G)	0	14(25%)	0
	484(P-H)	0	4(7.1%)	0
	486(I-T)	0	14(25%)	0

EBVaGCs, EBV-associated gastric carcinomas; NPCs, nasopharyngeal carcinomas; TWs, throat washings from healthy donors.

TAD: transactivation domain

NLS: nuclear localization signals

del: deletion

RG domain: arginine-glycine repeat motif domain

Amino acid: R is arginine, M is methionine, P is proline, T is theronine, Q is glutamine, K is lysine, G is glycine, I is isoleucine, L is leucine, E is glutamic, H is histidine

## Discussion

This is the first report on sequence intertypic polymorphism of the aa 148-487 in EBNA-2 gene in northern Chinese EBV A isolates. No sequence identical to the B95-8 prototype was found in any case, as three consistent changes were detected in all isolates (Figure 1). Of the three common mutations, amino acid mutation R163M and silent mutation c48998a were previously identified in 13 Korean isolates [24], 18 of 33 oral squamous cell carcinoma cases in Okinawa, Japan [25] and 6 human immunodeficiency virus-infected patients and the NPC samples in Hong Kong or Canton [26,27]. The silent mutation t49613a was detected in W91, an EBV A isolate from an African case of Burkitt's lymphoma [15] and GD1, from a southern Chinese patient [28]. The frequent mutation rate of these three positions in different geographical regions and different diseases suggested the mutational hot spots. A triplet insertion of ctc at nucleotide 49136 has been found in samples from German, US and African patients with a variety of nonmalignant and malignant disorders and W91 stain. A 51bp deletion at nucleotide 49102 was detected in LCLs from New Guinea as well as two German patients with fatal lymphoproliferative disease [29]. However, there were no changes in the nucleotide 49102 and 49136 in Northern China. Thus, these different changes of the EBNA-2 in different areas indicated the EBNA-2 variants geographically.

Frequent mutations in the analyzed sequences distinguished four subtypes of sequence variation in EBNA-2 gene. E2-A was the most common subtype in each population group. It was identified in 18 of 25 (72%) EBVaGC, 22 of 56 (39.3%) NPC and 16 of 32 (50%) TW isolates. Subtype E2-A was seen more in biopsies of EBVaGC samples and may be some association with EBVaGC. Moreover, more NPC isolates showed much more mutations than EBVaGC or healthy donors in E2-A (Figure 1). The E2-C is another dominant subtype in our study where two aa (357K, 358G) were deleted. Compared with the GD1 stain, which is a representative EBV strain isolated from a NPC patient in Guangdong, China [28], the deletions in the EBNA-2 domain was also detected in subtype E2-C. However, Walling DM et al [27] reported the EBNA-2 strain variation in oral hairy leukoplakia and did not identify these two mutations. Thus, whether the subtype E2-C was geographically specific or tumor preferential needs to be studied.

The subtype E2-D was detected only in the NPC samples and may be the subtype more relevant to NPC carcinogenesis. Further study with a larger number of samples is needed. Therefore, the NPC isolates were prone to harbor more variations and deletions in EBNA-2 gene than the other two groups (Figure 1 and Table 2), which is in accordance with our results from analysis on polymorphism of EBER and LMP2A [30,31]. It is conceivable that these variations in multiple viral genes are related to the persistence of EBV in NPC, which possibly contribute to the close association between virus and tumor.

As a transcription factor, EBNA-2 carries the TAD, NLS and a region which mediates promoter contact in aa 148-487 (Figure 2 and 3). The mutation R163M, which was located in the self-association (aa 148-214) domain of EBNA-2, was detected in all samples. The self-association domain was important for transcriptional activation and primary B-lymphocyte transformation [4]. This mutation was detected in oral hairy leukoplakia and the NPC in Hong Kong or Canton [26,27]. Then the mutation R163M may be a common mutation and have some effects on transformation of the EBNA-2 gene.

Characteristic features of EBNA-2 are a poly-proline and a poly-arginine-glycine (RG) stretch and conserved region (CR1-9) [28]. The CR5 (aa 296 to 309) make an important contribution to EBNA-2 transactivation function that mediates the contact between EBNA-2 and SKIP (Figure 2 and 3). The CR6 (aa 320 to 327) proved to be the CBF1 targeting domain which plays an important role in the B-cell immortalization (Figure 2 and 3) [29,30]. These two motifs were strictly conserved and verified their critical role in the maintenance of gene function.

The aa 357 and 358 belong to the RG domain of the EBNA-2 gene. EBNA-2 RG domain is a protein-protein and protein-nucleic acid interaction domain, important for efficient cell growth transformation, and a down-regulator of EBNA-2 activation of the LMP1 promoter [16]. Meanwhile, aa 357 to 363 (KGKSRDK) was the PKC phosphorylation site, which had effect in reducing the amounts of EBNA-2/CBF1 complex formed [32]. And these deletions were distributed in E2-C and E2-D subtypes (Figure 1). The deletions of aa 357K, 358G may be suggested to influence the EBNA-2 function. Therefore, whether the mutations in EBNA-2 gene affect its function is valuable to be investigated.

Aa 148-487 of EBNA-2 included two NLS domains and one TAD domain which were aa 284-341, aa 468-487 and aa 424-468 respectively. Simultaneously, for EBNA-2, one cytotoxic T-lymphocyte epitope has been defined: aa 276 to 291, associated with HLA-B18 [27]. The aa 291 change may alter the immune processing and recognition of the epitope in persons expressing HLA-B18 and possibly other HLA types. The nuclear localization loss of EBNA-2 function could facilitate the initiation of viral replication by failing to stimulate latency-associated gene expression and advantageous attribute in a permissive environment [27]. The mutations 291 and 294 were in NLS domain which may affect the nuclear localization of EBNA-2 function. The important role of EBNA-2 in transformation is likely to be in transcriptional regulation. An acidic transcriptional activation domain (AD) which can recruit an acitivation-related cell transcription factor and also bind EBNA-LP, enabling EBNA-LP to specifically coactivate transcription with EBNA-2 (Figure 3) [16]. The TAD (aa 424-468) and NLS (aa 468-487) were located in the AD domain in the carboxyl termini of EBNA-2 [16,17]. In this domain, residues (I438L, E476G, P484H and I486T) were only detected in the NPC samples (Figure 1 and Table 1) and the GD1 does not have these mutations. These mutations may be have some effect on the transcriptional regulation of EBNA-2 and suggest some association with NPC.

## Conclusions

In conclusion, we have classified 4 distinct subtypes of variation patterns in the EBNA-2 region coding aa 148-487 in EBV isolates of Northern China from multiple clinical specimens. The subtypes E2-A and E2-C were the dominant genotypes of the EBNA-2 gene in Northern China. Subtype E2-D was detected only in the NPC samples which may be associated with the tumorigenesis of NPC. Three variations (position 48991, 48998 and 49613) were detected in all of the tested samples which indicated a specific marker of EBV in Northern China. Mutation analysis in functional domains revealed NPC samples were prone to harbor more mutations than the other two groups. These results suggest that the EBNA-2 gene can be interesting to evaluate the association of EBNA-2 polymorphisms with EBV-associated tumors.

## Materials and methods

### Specimens and DNA extraction

In this study, 25 EBVaGCs, 56 NPCs and 32 TWs were used. Tumor tissues of GCs and NPCs were collected from the major hospitals of Shandong Province in the Northern China, a non-endemic area of NPC. The infection of EBV in GC and NPC tissues was determined by EBV-encoded small RNA (EBER) 1 in situ hybridization, as described previously [33]. TWs were collected from the healthy donors in the same geographic regions. The EBV-positive TWs were determined by the BamHI W fragment positive signals, using PCR with a BamHI W specific primer pair [34]. All the study subjects gave an informed consent for the study and the study was approved by the Medical Ethics Committee at the Medical College of Qingdao University, China.

DNAs from fresh specimens were extracted by using the standard method with proteinase K digestion and phenol-chloroform purification. QIAamp DNA FFPE Tissue kit (QIAGEN GmbH, Hilden, Germany) was used to extract the DNA from paraffin-embedded tumor tissues. All samples were EBV type A in our study.

### Amplification of DNA

The nested-polymerase chain reaction was used to amplify the DNA sequence coding aa 148-487 of the EBNA-2. The outer primers were EBNA-2-W/Y and the inner primers were EBNA-2-N/E (Table 3). The first PCR was performed in a total volume of 25 μl containing 1 × PCR reaction buffer, 100 ng of genomic DNA, 0.5 μM each primer, 200 μM of each deoxyribonucleotide triphosphates, and 1 U Pfu Taq polymerase (TaKaRa Biotechnology Co., Ltd., Kyoto, Japan). PCR amplification was performed with an initial denaturation at 94°C for 5min. Then, 35 cycles were carried out with denaturation at 94°C for 30 s, annealing at 53°C for 30 s, extension at 72°C for 1min. A final elongation step at 72°C for 10 min was also conducted.

**Table 3:** The sequence of the primers

Name	Sequence(5'-3')	Analyzed region(B95-8 coordinates)
EBNA-2-W1	GCTATGCGAATGCTTTGG	48891---48908
EBNA-2-Y1	GAGTCTTAGAGGGTTGCG	49512---49495
EBNA-2-N1	CTATGCGAATGCTTTGGA	48892---48909
EBNA-2-E1	TTGTTGGTCGTTGATGAC	49442---49425
EBNA-2-W2	AGAACCACGGTCCCCGACTGTA	49325---49346
EBNA-2-Y2	TGCTGAGAGCAAGGCACCAATT	50138---50117
EBNA-2-N2	ACGGTCCCCGACTGTATTTTAT	49331---49352
EBNA-2-E2	TTTTGGCAAGCCTTCCTT	50118---50101

W/Y were the outer primers; N/E were the inner primers

In each set of PCR, DNA from EBV-positive B95-8 cell lines, which isolated from a North American IM case, classified as a type A, was used as positive control, and nuclease-free distilled water served as negative control. The PCR products were analyzed by electrophoresis through a 1.2% agarose gel.

### Sequencing analysis of PCR products

PCR products were purified using a gel extraction kit (QIAEX II; QIAGEN GmbH, Hilden, Germany), under the conditions specified by the manufacturer. PCR amplified fragments were sequenced by means of a Prism ready reaction Dyedeoxy terminator cycle sequencing kit (Applied Biosystems, Foster, USA).

### Data analysis

The sequence data were checked for any homology in the NCBI sequence database by BLAST (National Center for Biotechnology Information; http://www.ncbi.nlm.nih.gov/) and were compared with the B95-8 prototype strain. Alignments between sequences were analyzed using DNA Star software (DNASTAR, Inc, version 7.0). Either χ2 test or Fisher's exact test (2-sided) was performed to determine the distribution difference of the EBV variations among the EBVaGCs, NPCs, and the TWs from the healthy adults. Significance was set at *P *value < 0.05. Statistical analyses were conducted using SPSS 17.0.

## List of abbreviations

EBV**: **Epstein-Barr virus; EBNA**-**2**: **The Epstein-Barr virus nuclear antigen 2; aa: amino acid; EBVaGCs: EBV-associated gastric carcinomas; NPCs: nasopharyngeal carcinomas; TWs: throat washings; BL: Burkitt's lymphoma; LMP: latent membrane protein; TAD: transactivation domain; NLS: nuclear localization signals; RG: arginine-glycine; CR: conserved regions; AD: acidic transcriptional activation domain.

## Competing interests

The authors declare that they have no competing interests.

## Authors' contributions

XYW carried out most of the studies and drafted the manuscript. YW and YC participated in parts of the studies and manuscript writing. XYW and GCW were responsible for the collection of specimens used in this study. ZFS and BL provided consultation and preparation of the final report. All authors read and approved the final manuscript.
